# Supplementary material for: Optimization of Conditions for Production of Soluble E. coli Poly(A)-Polymerase for Biotechnological Applications
Source: Biology (Basel). 2025 Jan 9;14(1):48. doi: 10.3390/biology14010048 (PMC11760895; doi:10.3390/biology14010048)
Supplement: Supplementary file 1 [file biology-14-00048-s001.zip › biology-3375053-supplementary.pdf]

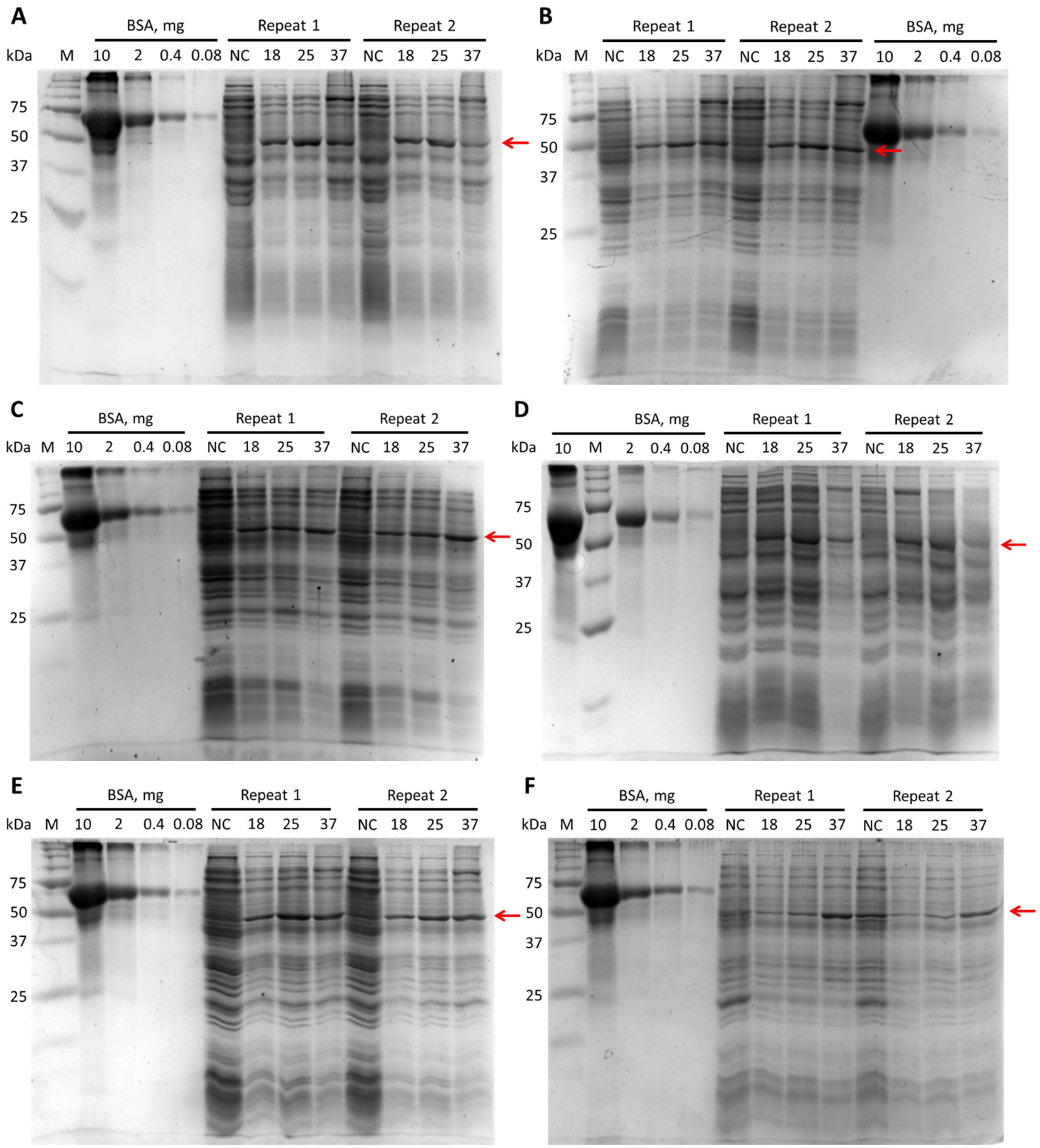
A
BSA, mg
Repeat 1
Repeat 2
kDa M 10 2 0.4 0.08 NC 18 25 37 NC 18 25 37
75
50
37
25
B
Repeat 1
Repeat 2
BSA, mg
kDa M NC 18 25 37 NC 18 25 37 10 2 0.4 0.08
75
50
37
25
C
BSA, mg
Repeat 1
Repeat 2
kDa M 10 2 0.4 0.08 NC 18 25 37 NC 18 25 37
75
50
37
25
D
BSA, mg
Repeat 1
Repeat 2
kDa 10 M 2 0.4 0.08 NC 18 25 37 NC 18 25 37
75
50
37
25
E
BSA, mg
Repeat 1
Repeat 2
kDa M 10 2 0.4 0.08 NC 18 25 37 NC 18 25 37
75
50
37
25
F
BSA, mg
Repeat 1
Repeat 2
kDa M 10 2 0.4 0.08 NC 18 25 37 NC 18 25 37
75
50
37
25

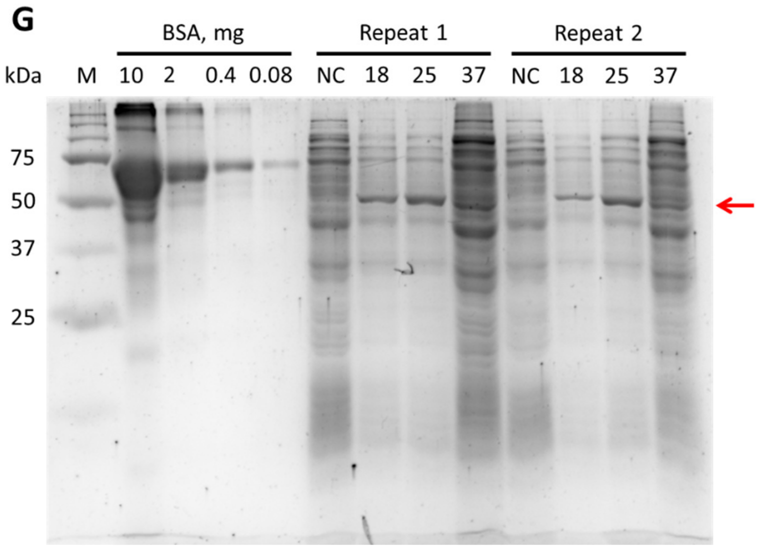

Supplementary Figure S1. Expression of recombinant *E. coli* PAP 1 in different *E. coli* strains. PAP 1 was expressed at 18, 25, 37°C in different *E. coli*, the resulting cell pellets were analyzed by SDS-PAGE. (**a**) — BL21 (DE3) pLysS, (**b**) — BL21 Gen-X, (**c**) — Lemo21 (DE3), (**d**) — SoluBL21, (**e**) — Rosetta 2 (DE3), (**f**) — Rosetta Blue (DE3), (**g**) — Shuffle T7. M — protein weight standard Precision Plus (Bio-Rad, Hercules, CA, USA); NC — negative control before PAP 1 expression; 18, 25, 37 — expression temperature, °C. BSA — amount of bovine serum albumin per ladder.

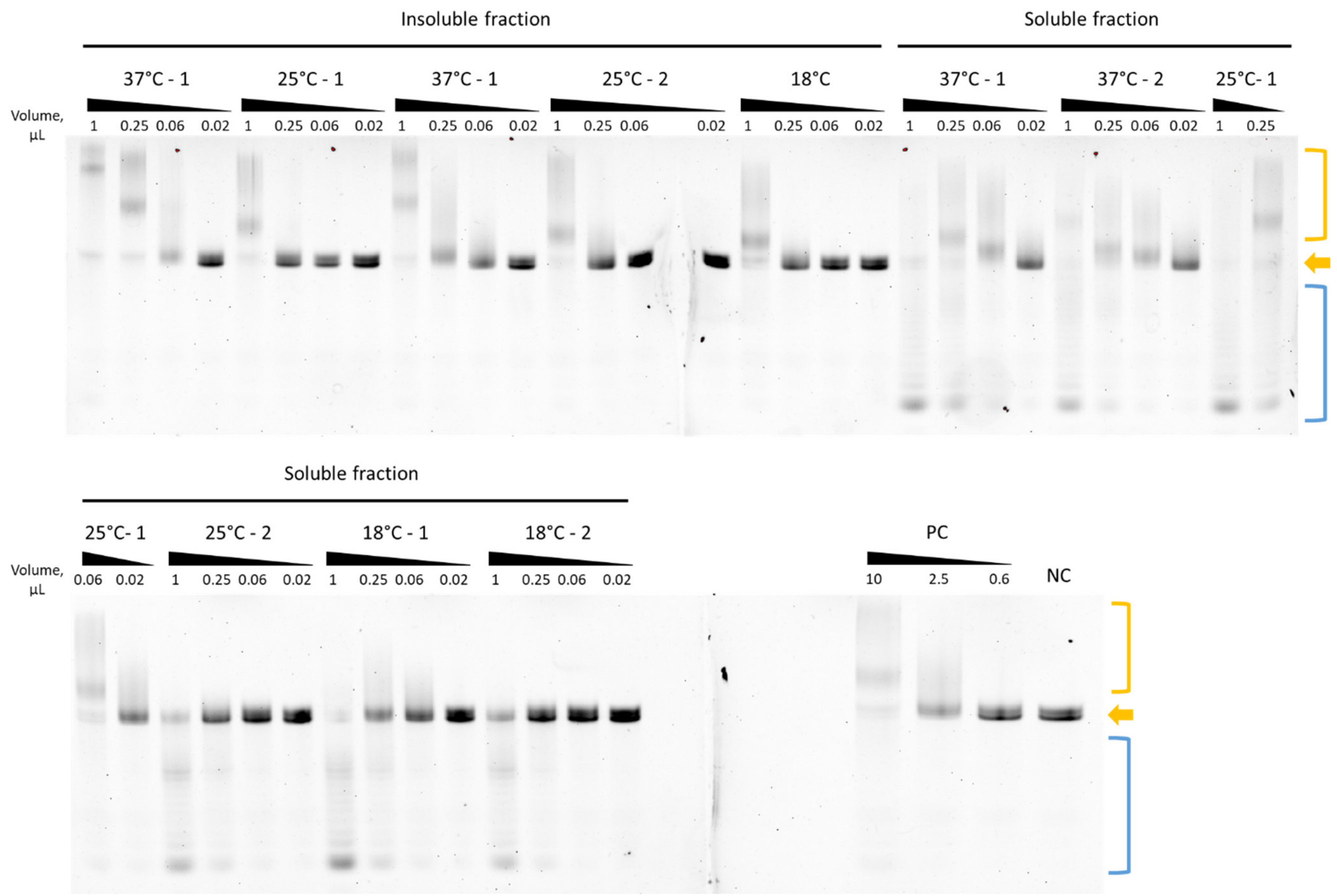

Supplementary Figure S2. Specific activity of recombinant PAP 1 after expression in Rosetta Blue (DE3). Specific activity of PAP 1 was measured using a fluorescently labeled (r)A20 substrate that was incubated with soluble and insoluble fractions of lysates. The reaction products were analyzed in denaturing PAGE. Elongated substrate is marked by an orange bracket, hydrolyzed substrate is indicated by a blue bracket, an orange arrow marks an unchanged substrate. Numbers above each lane denote a lysate volume; a fraction and an expression temperature are also given above lanes. PC means a commercial E. coli PAP 1 used as a positive control. NC marks a negative control (1 µL of the lysate per reaction) made from a control culture that did not contain the pET-PAP-Eco plasmid and was prepared identical to the cultures expressed recombinant PAP 1.
